# A Thesis on Tic Douloureux, or Neuralgia Faciei

**Published:** 1848-07

**Authors:** John McCalla

**Affiliations:** Philadelphia


### ARTICLE VII.

*A Thesis on Tic Douloureux, or Neuralgia Faciei.* Presented to the Faculty of the Baltimore College of Dental Surgery, by John McCalla, of Philadelphia, for the Degree of Doctor of Dental Surgery. Feb. 4, 1848.

The affection commonly called tic douloureux, or neuralgia, has its seat in the branches of those nerves that are distributed over the cheek, namely, the fifth and seventh pair of nerves, at the same time that no nerve in any part of the body is exempt from its attack, yet the fifth pair, or trifacial, seems to have been chosen as its favorite seat.

Proceeding as it does from that remarkable plexus which is bathed, as it were, in the blood of the cavernous sinus, and the branches of it passing through bony structures; the second and third, especially, being enveloped in bone to a great extent; it is, probably, from one or other of these anatomical circumstances, or from both of them combined, that the pain derives its peculiar character.

Its mode of attack differs in almost every case; in some the paroxysm commences with a dull obtuse pain not unlike an incipient tooth-ache, or some of the affections of the maxillary sinus, exciting at first, perhaps, little attention or alarm; gradually, however, it becomes more severe, and of more frequent recurrence, accompanied by acute, darting, plunging pains, shooting through the nerve affected, and the different branches of that nerve, with electrical rapidity, occasionally ceasing for a few moments as if for the purpose of rallying its scattered forces to renew the attack with redoubled energy; in this manner the unfortunate sufferer is thrown, as it were, into a state of frenzy. After having continued in this way for some time, the pain gradually subsides and the violent flashes cease for the time.

The pain attacks other persons suddenly and without warning, with an intensity of suffering which it is difficult to describe. In some cases the pain is characterised by a sensation of clawing and gnawing in the affected part, or as if a red hot iron was forcibly driven through the nerve, and accompanied by a repetition of agonizing shocks or flashes darting through it. The termination of the paroxysm is often as abrupt as the attack, whilst it leaves the patient either in a high state of nervous excitement, or completely exhausted by the agony. Sometimes the paroxysms intermit for the space of three or four days, or a week, or even a longer period, at other times they will return irregularly, several times in a day, setting at naught all prognosis, and obeying no regular laws; those that come on suddenly and violently from the first, are frequently the most severe, irregular and obstinate.

Cases are occasionally met with in which the paroxysms return three or four times every day in quite as regular order, as to the time, as they do in cases partaking of a more common or quotidian type. In others, again, two, three, or even four weeks may intervene between the paroxysms, yet the recurrence of them is so regular that the exact day on which they will return can be generally foretold. There is also a marked tendency in this disease to return annually about the same period. This is equally true of both forms, the regular and irregular. Some individuals assert that they suffer most in October and November, and others in April and May.

Tic douloureux in many of its phenomena is very analogous to epilepsy, hysteria, and other nervous diseases. In neuralgia, as in epilepsy, the parts are sometimes quiet, as, indeed, the whole system may be; yet there is so great a tendency to it that the slightest cause is sufficient to provoke an attack. Again, in neuralgia as in epilepsy the patient is occasionally forewarned of the approaching paroxysm by a feeling somewhat similar to the epileptic aura. We have heard of a case in which the paroxysm was invariably preceded by a sensation, as if a fine thread passed from about the region of the pylorus, through the chest and neck, to the seat of the pain in the cheek, and as soon as it reached that spot, the pain in the cheek began. Others have remarked that for some time previous to an attack, they invariably perceived a degree of nervous restlessness and irritability of temper, which rendered them unfit for any occupation either of business or pleasure, and made the society of their friends a burden to them. This state of feeling has been found to exist in cases depending on a disordered stomach. Among the various causes giving rise to this painful malady may be enumerated, dyspepsia, disease of the brain, disorder of the uterus, congestion of the liver and other viscera, morbid action of the spine, malaria. Local mechanical causes give rise to it, and in some persons there is found a constitution occasioning a neuralgic habit. Such, for example, are persons of a nervous and irritable disposition, who are morbidly sensitive to every impression both moral and physical, and on whom impressions produce more than ordinary corresponding effects. This extreme sensibility of the nervous system is found more frequently in females than males, and does not appear to arise so much from the irritation of any particular nerve, as from a peculiar idiosyncrasy. If, at any time, the feeble powers of such an one be further weakened by disease, an attack of pain having all the characteristics of true neuralgia will be the consequence.

We have thought proper thus to enumerate some of the causes of neuralgia, as well as the phenomena attending the disease, more for the purpose of showing the intimate relations existing between different and remote parts of the system, than from a desire to point out any particular mode of medical treatment. Our object at present is to consider some of the local mechanical causes giving rise to neuralgia, and endeavor to point out some of the surgical means to be employed in removing those causes.

Under the head of mechanical causes, or local irritation, may be mentioned, tumors growing in the tract of nerves, irritation of nerves from foreign substances, local injuries of nerves, exostosis, &c. The difficulty and embarrassment which have attended the diagnosis and treatment of these affections, have arisen principally from mistaken views of their pathology. They have too often been regarded as actual diseases of those nervous filaments which are the immediate seat of the neuralgia, instead of being considered as symptomatic of disease in the larger nervous masses from which those filaments are derived. This has given rise to the practice of dividing the second branch of the fifth pair of nerves where it passes out of the infra-orbiter foramen on the face, an operation which has deservedly gone into disrepute, as it is now generally admitted, that disease of the larger nervous masses, as the brain and spinal marrow, is not so much evinced by phenomena in the immediate seat of the disease, as in those more remote parts to which the nerves arising from the diseased portion are disturbed. That pain should be excited in nerves by irritation caused by tumors is not extraordinary; but it is difficult to account for pain in one nerve when another is irritated, in any other way than by the innumerable branches running in all directions, forming plexuses, and giving and receiving filaments from other aud remote branches. That such is the case there can be no doubt, as the following example will serve to show: it is the case of Lord ——, taken from the "Dublin Medical Press," reported by Sir

J. Murry, who states "that there was no symptom of tic douloureux for more than a year after the amputation of a leg, and not until the stump was injured by its being struck violently against a post, which produced excessive pain at the time and subsequently exfoliation of bone, since that time it appears the extremity of the nerve has become more irritable, and this has produced great constitutional irritation and disorder, which have apparently caused and kept up neuralgic pain in the cheek."

Several cases have been published of neuralgia arising from the encroachment of tumors on the course of nerves. When the tumor is large it cannot well escape detection, but occasionally it is so small as to be unnoticed by a careless observer, as is shown in the following case. Miss S. D. had been subject to neuralgia in the lower jaw for two years. When a physician was first called in, he found her considerably out of health and exhausted; the menstrual functions were performed regularly, although the secretion was of a pale dirty color. Having some defective teeth on the painful side of the face, they were removed with temporary relief. She was subsequently treated with tonics, and among others she took arsenic, which lessened the duration, but not regularity of the pain. On combing her hair she discovered a tender spot, which when pressed rather sharply gave her one of the severest attacks of pain in the jaw. On examination a tumor was found about the size of a horse-bean, situated on the parietal bone; pressure was again repeatedly tried and always produced the same severe pain; the tumor was removed with permanent relief, and speedy return to health.

Some persons have an extraordinary disposition to encysted tumors. Upwards of twenty have been met with in the same patient. The scalp seems more especially the seat of the atheromatous kind. These tumors are not painful, they are generally loosely situated just below the skin, and grow if left alone (particularly the steatomatous) to an enormous size. It is always best to extirpate them, as a troublesome sore is often the result of any attempt to promote suppuration. This operation may be performed by making an incision through the skin in the direction of the muscular fibres across the tumor and carefully dissecting it out. When the contents are of a fluid character, the yielding and floating condition of the walls of the cyst render the dissection very difficult. At this stage of the operation it has been recommended to open the cyst at its most depending part and expel its contents, after which the walls being in a collapsed condition may be more readily removed by the knife; the vessels supplying the tumor usually enter it at one point, and to prevent excessive hemorrhage may be reserved for the last stroke of the knife. They should now be taken up and secured by ligature, the coagulum removed, and the edges of the wound brought together and united by sutures or adhesive plaster, and then properly bandaged; the dressings to remain for several days. When the tumor is large, the double elliptical incision is indicated, as by this means we are enabled to remove a portion of the integuments which facilitates the operation, and cicatrization goes on more readily than when a redundance of integument is left.

Neuralgia has been known to follow the prick of a gooseberry thorn, attacking the patient with nervous symptoms, and impairing the general health which was only relieved by the amputation of the finger. In another instance neuralgia was cured by the removal of a fragment of china which had been lodged in the cheek for fourteen years.

The disease known as exostosis has long been classed among those giving rise to neuralgic affections, it is a bony excrescence or tumor growing out of, or upon, some part of a bone in consequence of an inflammatory action set up in the investing membrane. The morbid growth may arise, first from an osseous deposition seated between the external surface of the bone, and the internal surface of the periosteum, and adherent to both; secondly, from a similar formation originating in the medullary or lining membrane, and cancellated structure of a bone. Besides those just mentioned, Sir A. Cooper, describes two others, the cartilaginous, and the fungous exostosis; the first where the enlargement is preceded by the formation of cartilage, which forms the nidus for the ossific deposit; the second where the tumor is softer than cartilage, yet firmer than fungus in other parts of the body, containing spiculæ of bone. It is usually known as osteo-sarcoma. That this disease sometimes depends upon local irritation there can be no doubt, as we have on record several well authenticated cases of osteo-sarcoma of the maxillary bones, which were completely cured by the extraction of the neighboring teeth and the subsequent exfoliation of a portion of the bone, and alveolar processes; the great majority of cases, however, will be found to depend upon some constitutional taint, and then we have little to hope for from any mode of treatment that may be adopted: even amputation is no certain cure, as the disease is liable to occur in another situation.

An exostosis when not very large, causes but little inconvenience, but when it has extended itself, the surrounding soft parts become affected, the muscles stretched, the cellular substance thickened, and the functions of the nerves and arteries interrupted; in short, wherever these enlargements occur in the neighborhood of important organs, and increase to any size, a loss of power must be experienced in their functions; when this disease occurs in the teeth it is always on the roots, and in some cases where the growth is not rapid, it may exist for considerable length without causing the patient much uneasiness. As the fang becomes enlarged, however, the patient experiences a dull continued pain in the affected tooth, yet probably not so severe as to determine him upon its extraction, but when the deposition of bone is more rapid, or the walls of the alveolus less yielding, the pain is more acute and lancinating, resembling that of tic douloureux. The pain now extends, not only to the contiguous teeth, but to the jaw, cheek, and forehead, shooting along the different branches of the fifth pair of nerves with the rapidity of lightning. This is frequently so intense as to mask the immediate cause of it, and render its discovery exceedingly difficult; in forming a diagnosis it will be necessary to examine the teeth carefully, which may be done by striking the tooth or teeth suspected, with the handle of an instrument.

As the investing membrane will be found very much inflamed and highly sensitive, a blow given upon the side of the tooth on which the disease is situated will be followed by severe pain and thus discover the seat and nature of the disease. It may exist when the crowns are healthy, but it more frequently manifests itself upon subjects whose teeth have become painful, either from indolent inflammation, caused by the decay of the body of the tooth, and which extends even to the roots, or the result of a gouty or rheumatic diathesis. Numerous cases of neuralgia of the face have occurred, which, resisting for a length of time the usual constitutional remedies, have been traced to this source and immediately relieved. In dental exostosis nothing remains to be done but the extraction of such teeth as are affected, and the sooner this is done after it has been discovered to exist, the better, for although the alveolar cavity may increase in size as the osseous formation progresses, the opening toward the gum is seldom, if ever, enlarged so as to admit of the extraction of the tooth with facility. To render the operation more safe, the gum should be freely dissected away from the tooth by making a vertical incision through it and dissecting it back to each side. The portion of alveolus in connection with the exostosed fang may now be removed, which will afford an easy exit for the tooth and render the liability to fracture the jaw, much less than when such precautions are omitted.

The treatment of tic douloureux must differ as widely in different cases as the causes that give rise to it. In those cases in which the disease assumes the intermittent type, it can be relieved, as all other cases of intermittent and periodical disease, by the exhibition of quinine, or arsenic. If the pain can be traced to an impaired state of the digestive organs, a course of medicine calculated to restore them to a healthy condition is clearly indicated. When neuralgic symptoms can be traced to a morbid state of some portion of the spinal marrow, the treatment consists in local depletion by leeching or cupping, and counter irritation by blisters to the affected portion of the spine, and in very obstinate cases issues or setons have been used with decided advantage.

Baron Larrey, surgeon general to the army of Napoleon, recommends the moxa in neuralgic affections of a chronic character; he says, "when convulsive and habitual motions of certain muscles (which characterise tic douloureux) have become chronic, whatever may be the cause; or are the result of a mechanical cause, which weakens the tissue of the nerves of the muscles, the moxa is perfectly indicated; but it should be applied as near as possible to the seat of the disease, and upon the course of the injured nerves. The lesion consists in the chronic engorgement and inflammation of the membrane which invests the nerves of the affected part. This remedy, by producing an excitation upon these organs, causes a salutary derivation of the morbid principle, and re-establishes a healthy action.

"It is not equally indicated in acute neuralgia, proceeding from spontaneous causes, nor in tetanic affections, because it augments the irritation and tetanus."

When neuralgia faciei depends on some peculiarity of constitution, the pains arise spontaneously as it were, whenever the system is reduced to a state of general debility; in others in whom this morbid state is not so highly developed, yet exists to an extent sufficient to predispose them to these pains, it requires only a very trifling cause of irritation, or a temporary or slight derangement of the general health to produce them.

The nature and peculiarity of each case must be carefully studied and treated accordingly, bearing in mind that females of such habits are incapable of enduring harsh or violent remedies, but that a steady and patient perseverance with mild ones is necessary until a more healthy action is induced, then by a cautious use of tonics combined with sedatives, and a proper attention to regimen, the general health may be so improved that the neuralgic habit will at length, to a certain extent, be overcome.

